# MDSCs in breast cancer: an important enabler of tumor progression and an emerging therapeutic target

**DOI:** 10.3389/fimmu.2023.1199273

**Published:** 2023-07-03

**Authors:** Haoyu Liu, Zhicheng Wang, Yuntao Zhou, Yanming Yang

**Affiliations:** ^1^ Department of Radiotherapy, Second Hospital of Jilin University, Changchun, China; ^2^ National Health Commission (NHC) Key Laboratory of Radiobiology, School of Public Health, Jilin University, Changchun, China

**Keywords:** myeloid-derived suppressor cells, breast cancer, immunosuppression, immunotherapy, tumor microenvironments

## Abstract

Women worldwide are more likely to develop breast cancer (BC) than any other type of cancer. The treatment of BC depends on the subtype and stage of the cancer, such as surgery, radiotherapy, chemotherapy, and immunotherapy. Although significant progress has been made in recent years, advanced or metastatic BC presents a poor prognosis, due to drug resistance and recurrences. During embryonic development, myeloid-derived suppressor cells (MDSCs) develop that suppress the immune system. By inhibiting anti-immune effects and promoting non-immune mechanisms such as tumor cell stemness, epithelial-mesenchymal transformation (EMT) and angiogenesis, MDSCs effectively promote tumor growth and metastasis. In various BC models, peripheral tissues, and tumor microenvironments (TME), MDSCs have been found to amplification. Clinical progression or poor prognosis are strongly associated with increased MDSCs. In this review, we describe the activation, recruitment, and differentiation of MDSCs production in BC, the involvement of MDSCs in BC progression, and the clinical characteristics of MDSCs as a potential BC therapy target.

## 1 Introduction

Breast cancer (BC) is the primary driver of cancer-related mortality among females globally (1). BC can be categorized into four different subtypes based on the expression of the Human epidermal growth factor receptor (HER2/neu), the estrogen receptor (ER), and the progesterone receptor (PR): triple negative breast cancer (TNBC), Luminal A, Luminal B, and HER2 (+). This classification is the basis for most BC treatments, including surgery, radiotherapy (RT), chemotherapy, targeted therapy, and immunotherapy (2). Immunotherapy is becoming increasingly essential in treating other tumors, but only a tiny percentage of BC patients are aid from immunotherapy at present. In fact, the therapeutic relevance of immunotherapy is restricted to a minority of patients, and secondary resistance further limits the effect of immunotherapy to substantially better the prognosis of BC patients (3). Immunotherapies, particularly immune checkpoint inhibitors (ICIs), are ineffective as a sole treatment in most patients with BC. Tumor microenvironment (TME) is widely accepted as having an irreplaceable function in the drug resistance mechanism. Additionally, Myeloid-derived suppressor cells (MDSCs) serve an essential function in TME (3).

Myeloid-derived suppressor cells (MDSCs), a diverse group of immature myeloid cells (IMCs), serve an essential function in the immune cell network (4). MDSCs play a role in tumor development via a variety of immunosuppressive mechanisms, including metabolite depletion, upregulation of reactive oxygen species (ROS), and secretion of multiple cytokines, and kinds of non-immunosuppressive mechanisms, like epithelial-mesenchymal transformation (EMT), promoting tumor cell stemness, as well as tumor vascular production (5). Previous research has demonstrated a correlation between MDSCs and poor overall survival (OS) in patients (6). Consequently, cancer immunotherapy can be proven to enhance by fostering MDSCs elimination, inhibiting their recruitment, expansion, and function.

In this review, we summarize the immunosuppressive function of MDSCs, their role in BC, and strategies for targeting them in various cancer treatments. We explore the feasibility and prospective development of multiple targeted MDSCs detection methods and therapeutics for the treatment or prevention of BC.

## 2 MDSCs in breast cancer

### 2.1 Classification of MDSCs in breast cancer

MDSCs from BC patients are functionally and phenotypically similar to MDSCs that come from bone marrow, indicating that MDSCs emerge from myeloid precursors (4). Granulocytic (G)-MDSCs (CD11b^+^Ly6G^+^Ly6C^low^) and monocytic (M)-MDSCs (CD11b^+^Ly6G^-^Ly6C^high^) could be precisely identified relying on their phenotypic and morphological characteristics in mice as research demonstrates (7). In contrast to mouse MDSCs, isolating and identifying human MDSCs is difficult due to their heterogeneity, proximity phenotypic and functional closeness to other cell subpopulations, and lack of definitive markers (7, 8). MDSCs are correspondingly classified into two categories in humans. CD11b^+^CD33^+^HLA^-^DR^-/low^ CD14^+^CD15^-^ identifies M-MDSCs, whereas CD11b^+^CD33^+^HLA^-^DR^-/low^CD14^-^CD15^+^(or CD66b^+^) identifies G-MDSCs (6, 7). The identification of CD84 as MSDC-specific cell surface markers in BC was reported using a single-cell transcriptomic approach by Alshetaiwi et al. (9). In addition, a subpopulation of MDSCs in humans is comprised of undifferentiated progenitor cells known as e-MDSCs, identified as HLA-DR^-^CD33^+^Lin^-^ (CD3/14/15/19/56) for which the mouse counterpart has not been discovered (7) (**Table 1**).

**Table 1:** Phenotypic characteristics necessary to identify cells as MDSC.

	Human	Mouse
G-MDSCs	CD11b^+^CD33^+^HLA-DR^-/low^CD14^-^CD15^+^(or CD66b^+^)	CD11b^+^Ly6G^+^Ly6C^low^
M-MDSCs	CD11b^+^CD33^+^HLA-DR^-/low^ CD14^+^CD15^-^	CD11b^+^Ly6G^-^Ly6C^high^
e-MDSCs	HLA-DR^-^CD33^+^Lin^-^ (CD3/14/15/19/56)	Not clearly determined

MDSCs, myeloid-derived suppressor cells; G-MDSCs, polymorphonuclear-MDSCs; e-MDSCs, early-stage MDSCs; M-MDSCs, monocytic-MDSCs.

While phenotype is a necessary step in defining MDSCs, please note that the identification of MDSCs cannot be based on immunophenotype alone, but requires proof of their lymphocyte suppressive function.

### 2.2 MDSCs production and activation in breast cancer

The development of MDSCs is governed by a complicated system of signals that can be categorized into two groups: those that promote the accumulation of IMCs and those that cause the activation of these cells (10). The signal transducer and activator of transcription (STAT) family, IFN regulators, Notch, adenosine receptor A2b, NLRP3, and many other signaling pathways and regulators are responsible for stimulating myelopoiesis, inhibiting the maturation and differentiation of progenitor cells, and promoting the expansion of the IMCs (9, 10). The other group is accountable for the pathological activation of immature cells that acquire an immunosuppressive phenotype, involving diverse signaling pathways and regulators such as NF-κB pathway, STAT1 pathway, STAT6 pathway, prostaglandin E2 (PGE2), and Cyclooxygenase-2 (COX-2) and ER stress response pathway (9, 10). These two groups of signals partially overlap but are controlled by various transcription factors and intermediates, both of which are necessary for the accumulation of MDSCs.

**Figure 1:**
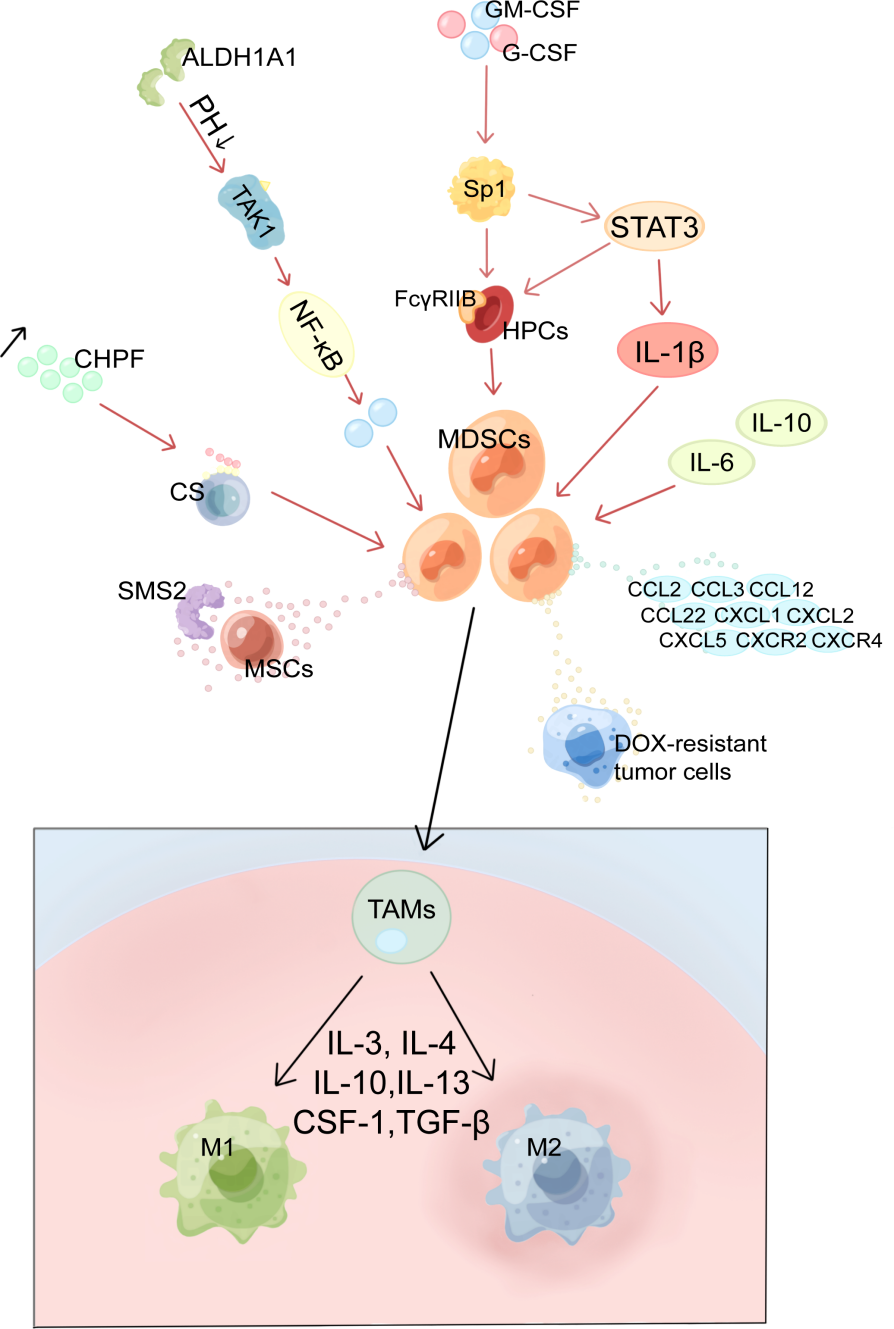
Mechanism of MDSCs production and activation, recruitment and differentiation in BC. MDSCs, myeloid-derived suppressor cells; HPCs, haematopoietic progenitor cells; Sp1, specific protein 1; GM-CSF, granulocyte-macrophage colony-stimulating factor; G-CSF, granulocyte colony-stimulating factor; ALDH1A1, aldehyde dehydrogenase 1A1; TAK1, TGF-β-activated kinase 1; CS, Chondroitin sulfate; CHPF, chondroitin polymerase factor; SMS2, Sphingosine synthase 2; MSCs, mesenchymal stem cells; TAMs, tumor-associated macrophages; M1, M1 macrophages; M2, M2 macrophages.

In BC, factors that contribute to the amplification and activation of MDSCs involve granulocyte-macrophage colony-stimulating factor (GM-CSF) (10, 11), granulocyte colony-stimulating factor (G-CSF) (11, 12), PGE2 (13, 14), vascular endothelial growth factor (VEGF) and interleukins (IL-1 (15), IL-6 (16), IL-13 (17), IL-17 (18), IL-20 (19), IL-33 (20), IL-34 (21)), macrophage migration inhibitory factor (MIF) (22), microRNAs (miRNAs) derived from tumor exosomes (23). It is interesting to note that higher levels of psychological stress in patients with BC might result in the production of stress-related hormones and cytokines (IL-1Rα, IP 10, G-CSF, and IL-6), which in turn stimulate the production and accumulation of MDSCs (24) (**Figure 1**).

Tumor cell derived G-CSF and GM-CSF are key factors contributing to MDSCs accumulation (10, 11). CSF raises the levels of FcγRIIB on hematopoietic progenitor cells (HPCs) by activating specific protein 1 (Sp1), which then activates the STAT3 signaling pathway, which biases hematopoiesis toward the myeloid lineage spectrum and promotes MDSCs production by HPCs (10, 25). Moreover, chondroitin polymerase factor (CHPF), which is frequently highly expressed in BC tissues, facilitates the binding of G-CSF to cell surface Chondroitin sulfate (CS) and promotes the accumulation of MDSCs (26). In addition, aldehyde dehydrogenase 1A1 (ALDH1A1) relies on its enzymatic activity to reduce intracellular pH in BC cells in order to increase TGF-β-activated kinase 1(TAK1) phosphorylation and activate NF-κB pathways, which then induces GM-CSF secretion, thereby inducing MDSCs amplification and promoting BC progression (27).

With a diameter of 50–200 nm, exosomes are lipid bilayer structures that are important in the reprogramming of TME. IL-6, IL-10, and other cytokines released by BC cells (4T1) exosomes would boost the stimulation and expansion of MDSCs by promoting STAT3 phosphorylation in myeloid cells, which would, in turn, reduce myeloid proliferation and death and hasten their differentiation into MDSCs (23). Jiang M et al. used miRNA microarrays to identify miRNAs secreted by tumor exosomes; two of them, miR-9 and miR-181a, were shown to target protein inhibitors of activated STAT3 (PIAS3) and cytokine signaling protein 3 (SOCS3), respectively, therefore activating the JAK/STAT signaling cascade (28). Moreover, the accumulation of early-stage MDSCs was stimulated by prolonged SOCS3 suppression and aberrant upregulating of the JAK/STAT signaling pathway (29).

In addition, various pathway signaling molecules overlap with each other and can form loops to regulate each other. In BC cells Mammalian target of rapamycin (mTOR) signaling stimulates MDSCs accumulation by regulating G-CSF, and MDSCs were found to mutually increase Tumor-initiating cells frequency by activating Notch in tumor cells, which in turn promotes G-CSF secretion, forming a feed-forward loop leading to further MDSCs expansion (30). Furthermore, by inducing autocrine secretion of GM-CSF, IL-33 in the TME inhibits apoptosis and maintains MDSCs survival, resulting in a positive feedback cycle for MDSCs accumulation (20).

### 2.3 MDSCs recruitment in breast cancer

Many different variables can induce the recruitment of MDSCs into tumor tissue. These factors include chemokines, cytokines, and complements generated by tumor cells and normal cells. Chemokines are an essential component in the process of recruiting MDSCs (31).

BC regulates the production of chemokines by MDSCs via multiple pathways. Enhanced lung fibroblast CXCL1 secretion reduces the immunity of lung microenvironment by recruiting G-MDSCs and facilitates the formation of niches in the anterior lung metastases of BC (32). Inactivation of retinoblastoma enhances the secretion of the chemoattractant CCL2. Consequently, the activated CCL2-CCR2 axis in the TME promotes tumor angiogenesis and recruitment of tumor-associated macrophages (TAMs) and MDSCs to the TME (33). BC exosomes convey upregulated miR-200b-3p, taken by alveolar epithelial type II cells, and targets PTEN straightforwardly. Suppression of PTEN facilitates activation of the AKT/NF-κBp65 pathway, which increases CCL2 expression and MDSCs recruitment, thereby promoting pulmonary metastasis of BC (34). ERO1-α, a tumor-associated endoplasmic reticulum disulfide oxidase, facilitates the oxidative folding of G-CSF, CXCL1, and CXCL2 to produce and recruit G-MDSCs (35). TGF-β1 promotes miR-494 upregulation in MDSCs, and miR-494 expression enhances CXCR4-mediated MDSCs chemotaxis (36). The transcription factor ΔNp63 has been reported to directly regulate CXCL2 and CCL22 to promote MDSCs recruitment in TNBC (37).

A diversity of other elements also influences the recruitment of MDSCs. S100A8/A9 proteins are chemotactic for MDSCs; for instance, the lung and liver can express high levels of S100A8, which promotes the recruitment of MDSCs at these sites, thereby promoting BC metastasis (38). Chen JY et al. found that BC-derived VEGF-C upregulates chemokines derived from lymphatic endothelial cells (LECs) to recruit MDSCs to TME and lymph nodes (LNs) via CXCR2 (39). There is experimental evidence that, in coordination with LECs, interstitial fluid flow promotes the spread of MDSCs at the existence of 4T1 cells and that inhibition of VEGFR 3 reduces the flow response of MDSCs and 4T1 cells (40). Moreover, epigenetic regulation is implicated. The epigenetic regulator Lysine acetyltransferase 6A (KAT6A) dependent SMAD3 protein acetylation promotes MDSCs recruitment and TNBC metastasis (41). In addition, complement activation and C5a signaling are involved in the recruitment of MDSCs into TME and in the inhibition of CD8^+^ T cell mediated tumor elimination, thereby inducing angiogenesis in the lungs of tumor-bearing mice, promoting BC lung metastasis (42). Interestingly, Cheng R et al. discovered that periodontal inflammation (PI) could upregulate CCL5, CXCL12, CCL2, and CCL5. These chemokines recruit MDSCs, which can generate pre-metastatic ecological niches at inflammation sites to promote the metastasis of BC (43).

### 2.4 MDSCs differentiation in breast cancer

MDSCs can differentiate under the regulation of multiple transcription factors (**Figure 1**). They differentiate into TAMs and inflammatory dendritic cells (inf-DCs). Since G-MDSCs are typically have a brief half-life and differentiate into tumor-associated neutrophils (TAN), the differentiation of M-MDSCs has been studied in more detail (44). In response to environmental stresses such as hypoxia, M-MDSCs can differentiate into TAMs after migrating to the target tissue and undergoing migration. In response to lipopolysaccharide (LPS), TNF-α, and IFN-γ, TAMs can polarize into either the M1 phenotype, which has pro-inflammatory and anti-tumor activities, or the M2 phenotype, which has pro-tumor activities (45).

Development and progression of BC are accompanied by a transition from MDSCs to TAMs. In MDSCs, IL-33 induces the expression of IL-13 while suppressing that of IL-12. As a result, M2 macrophages and Th2 cells may become polarized inside the tumor site, which is detrimental to anti-tumor immunity (20). Similarly both Sphingosine synthase 2 (SMS2) and exosomes secreted by mesenchymal stem cells (MSCs) can speed the progression of BC by inducing the differentiation of M-MDSCs into immunosuppressive M2-polarized macrophages (45, 46). Tumor cells resistant to doxorubicin (DOX) secrete PGE2, which stimulates the EP2-EP4/cAMP/PKA signaling pathway in MDSCs and, in turn, increases MDSCs growth and M2 polarization by inducing miR-10a expression (13). In addition, Natural killer T (NKT) cells have been shown to promote the transformation of CD11b^+^ HLA-DR^-^MDSCs into CD11b ^low^ HLA-DR dendritic cells (DCs) (47).

### 2.5 Identification and detection of MDSCs in breast cancer

The current methods commonly used for MDSCs detection are magnetic selective enrichment, sorting and purification, flow cytometry sorting, and density gradient separation (48). MDSCs identification and functional assays are aided by the secretion of downstream molecules and the determination of target cell effects. Recently several novel assays have been proposed. Hoffmann SHL et al. demonstrated that targeting MDSCs cell surface integrin CD11b with radionuclide-labeled monoclonal antibody (mAb) has little effect on cell viability and function, and can be used to image MDSCs in tumor-bearing mice using PET (49). Sceneay J et al. discovered that BM-MDSC (GM-CSF and IL-6 cultured bone marrow cells to produce MDSCs nearly identical to G-MDSCs) could be persistently tagged with a near-infrared fluorescent dye called DiD and monitored *in vivo* by optical imaging (OI), as well as repositioned and identified *in vitro* by flow cytometry. This technique permits the study of the distribution and destiny of DiD-labeled BM-MDSC in breast tumor-bearing mice as a way of finding MDSCs in BC that present new organ-specific changes (50). Although there have been many attempts of targeted detection methods for MDSCs, most of them can only stay in the laboratory stage and still have problems of complex methods and confusing detection. There is still a need to develop more rapid functional assays as well as definitive marker molecular assays.

## 3 Immunosuppressive effects and non-immune functions of MDSCs in the progression of breast cancer

**Figure 2:**
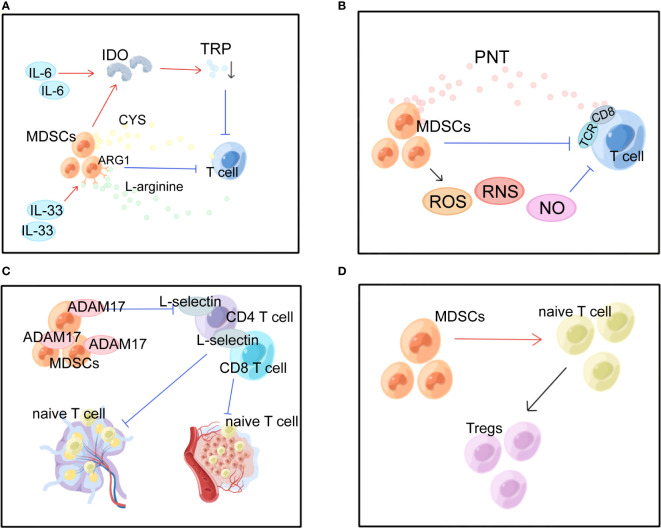
Multiple immunosuppressive effects of MDSCs in BC. MDSCs, myeloid-derived suppressor cells; IDO, indoleamine 2, 3-dioxygenase; TRP, tryptophan; ARG1, arginase 1; CYS, cysteine; PNT, peroxynitrite; TCR, T cell receptor; ROS, reactive oxygen species; RNS, reactive nitrogen species; ADAM17, a disintegrin and metalloproteinase domain 17. **(A)** MDSCs suppress T cells by depleting nutrients such as ARG, TRP, and CYS. **(B)** MDSCs produce ROS, RNS and NO causing oxidative stress and suppressing T cells. **(C)** MDSCs interfere with lymphocyte migration through direct contact and plasma membrane expression of ADAM17.**(D)** MDSCs promote the conversion of naive CD4^+^ T cells into Tregs.

**Figure 3:**
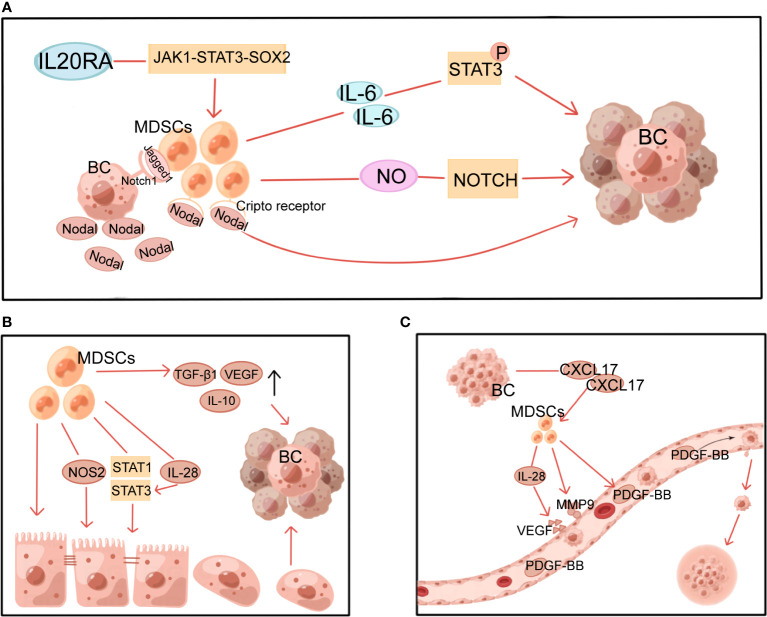
Multiple non-immune functions of MDSCs in BC. MDSCs, myeloid-derived suppressor cells; NOS2, nitric oxide synthase 2; VEGF, vascular endothelial growth factor; PDGF-BB, platelet-derived growth factor-BB; MMP9, matrix metalloproteinase 9; BC, breast cancer. **(A)** MDSCs induce and maintain tumor cell stemness through STAT3 and Notch pathways. **(B)** MDSCs induce EMT through multiple mechanisms. **(C)** MDSCs induce angiogenesis in tumors.

MDSCs substantially suppress tumor-fighting T and B cells, particularly cytotoxic T lymphocytes (CTL) and pro-inflammatory cells like natural killer (NK) cells in TME. Additionally, MDSCs support cancer progression by inducing Tregs and Th17 cells, therefore modifying the microenvironment that fosters tumor development and driving cells to evade immune surveillance (51) (**Figure 2**). In addition, MDSCs also promote BC progression through non-immunosuppressive pathways including promotion of tumor stem cells, mediation of EMT, and promotion of angiogenesis. These functions are summarized in **Figure 3**.

### 3.1 Immunosuppressive effects of MDSCs

In the BC microenvironment, MDSCs exert immunosuppressive effects in several ways. The most prominent of these is the suppression of T-cell activity. The first mechanism is nutrient depletion (**Figure 2A**). MDSCs have been demonstrated to conduct their suppressive function by stimulating the formation of indoleamine 2, 3-dioxygenase (IDO), which diminishes local tryptophan (TRP) and creates cytotoxic metabolites such as kynurenine in the TME and lymphatic drainage areas, which causes a rise in Tregs, inhibition of antigen-specific immune responses, and suppression of tumor-specific CTLs (52). Overexpression of IDO is maintained by STAT3-dependent NF-κB activation by IL-6 (16, 52). Moreover, IL-33 strengthened the suppressive action of MDSCs against T cells arginase 1 (ARG1)-dependent L-arginine depletion (19, 50). The cysteine (CYS) that T cells need for activation and proper functioning is depleted because MDSCs devour CYS but do not return CYS to their surroundings (53).

The second mechanism is oxidative stress generation (**Figure 2B**). MDSCs can inhibit T cells in the TME via ROS, reactive nitrogen species (RNS), and nitric oxide (NO). MDSCs induce tolerance in T cells through nitration/nitrosylation of T cell receptor (TCR) and CD8^+^ molecules on the cell surface and production of the free radical peroxynitrite (PNT) (54). Stiff A et al. found that MDSCs also create NO to inhibit NK cell FcR-mediated activity, which lowers the effectiveness of mAb treatment and hinders anti-tumor immunity (55).

The third mechanism is the one that inhibits lymphocyte migration (**Figure 2C**). In peripheral lymphoid organs, MDSCs reduce immunological response and aggregate in sentinel LNs, where they block CD3/CD28-induced T cell reproduction in a contact-dependent way. And this facilitates tumor development and metastasis (50, 55). Hanson EM et al. discovered that by downregulating L-selectin expression on the surface of CD4^+^ and CD8^+^ T cells via plasma membrane production of ADAM17 (a disintegrin and metalloproteinase domain 17), MDSCs in BC restrict the activation and entrance of naive T cells into LNs and the transport to tumors, ultimately suppressing anti-tumor immunity (56).

The fourth is the expansion mechanism and activation of Tregs. MDSCs promote the expansion and transformation of naive CD4^+^ T cells into Tregs (**Figure 2D**). However, the mechanism is not entirely comprehended. Tumor-infiltrating Tregs are important factors in antitumor immunosuppression. Inducible interleukin-34 (IL-34) causes the conversion of bone marrow stem cells (BMSCs) into M-MDSCs, which indirectly attenuates the immune response by producing CCL22-recruiting Tregs in TME, causing chemoresistance (21). In addition, BC-induced MDSCs can induce effector T cells and convert them into Tregs through the IDO mechanism (52). It has been established that T cells and MDSCs have a retaliatory interaction, with MDSCs suppressing T cell activation while stimulated T cells mediating MDSC death via the Fas-FasL pathway (57).

MDSCs in BC can also act by suppressing other immune cells. MDSCs can act through contact-dependent mechanisms and also indirectly by secreting NO, ARG, and IL-1 to suppress anti-tumor B cell responses (58). MDSCs can convert normal B cells into immunomodulatory B cell (Bregs) subtypes that suppress T cell responses (59). Furthermore, the expression of programmed cell death protein 1 (PD-1) is substantially upregulated in MDSCs, and studies have implicated various mediators in TME, like LPS, in inducing PD-1 expression in MDSCs in BC (60). Via the PD-1 and programmed cell death ligand 1 (PD-L1) axis, MDSCs have been demonstrated to increase PD-1/PD-L1 Bregs-mediated immune evasion by activating the phosphatidylinositol 3-kinase (PI3K)/protein kinase B (AKT)/NF-κB signaling pathway in B cells (61). In addition, medroxyprogesterone acetate, a progesterone analog, promotes MDSCs in the 4T1 tumor model to inhibit NK cell degranulation and IFN-γ production by inducing IDO production (62). BC cells grown under hypoxia release kinds of cytokines such as monocyte chemotactic protein-1, attract MDSCs and decrease the cytotoxic effects of NK cells, all of which foster the metastasis of cancer (63). Nevertheless, NKT cells may rescue suppressed T cells by transforming CD11b^+^ HLA-DR^−^ MDSCs into CD11b^low^ HLA-DR DCs through an NKG2D-dependent signaling mechanism (47). C5aR signaling contributes to MDSCs function by regulating CD4^+^ T cells are polarized to Th2 type in the lungs of tumor-bearing mice (42). In the lungs of tumor-bearing mice, treatment with DOX was shown to increase miR-126 exosomes from MDSCs, which in turn contributed to MDSCs mediated inhibition of Th1 cell activation, suppression of T cell activity, and induction of Th2 cell responses (64).

### 3.2 Non-immune functions of MDSCs

#### 3.2.1 Induction and maintenance of stemness in tumor cells

By generating and sustaining cancer stem cells(CSCs), MDSCs provide a non-immune role that promotes tumor development and metastasis. This process involves multiple mechanisms, with STAT3 and Notch playing a significant role (**Figure 3A**). It has been demonstrated that MDSCs-derived IL-6 initiates STAT3 phosphorylation and that MDSCs-derived NO activates NOTCH, which then cooperates with IL-6 to cause sustained STAT3 activation (65). This suggests that MDSCs, via interacting with IL-6/STAT3 and NO/Notch, could contribute to activating and sustaining the pool of CSCs (65). In addition, the Janus kinase 1 (JAK1)-STAT3-SOX2 pathway was also discovered to be involved in the effects of IL-20RA on BC cell stemness and the promotion of more MDSCs (19). Sprouse ML et al. found that through activating the nuclear factor E2-related factor 2 (NRF2)-antioxidant response element axis, G-MDSCs increase ROS generation, which in turn increases Notch1 receptor expression in circulating tumor cells (CTCs). Direct cell contact between Jagged1-expressing G-MDSCs and Notch1 receptors on CTCs promotes Nodal secretion in Notch-activated CTCs. Nodal induces pro-tumor differentiation of G-MDSCs by binding to Cripto receptors abundantly expressed in G-MDSCs, thereby promoting CTCs survival and proliferation and BC metastasis (66). Furthermore, there is experimental evidence that MDSCs express pro-metastatic factors, including chitinase 3-like 1 and matrix metalloproteinase 9 (MMP9) in a ΔNp63-dependent manner to promote CSCs function in TNBC and facilitate TNBC metastasis (37).

#### 3.2.2 Epithelial-mesenchymal transition

EMT is the process through which epithelial cells shed their adherent phenotypes and take on more mesenchymal features increasing cell mobility. This process has been demonstrated to be essential in tumor progression, from initiation to metastasis. MDSCs can facilitate this process (**Figure 3B**). It has been demonstrated that surgical stress shortens the OS of mice and raises the number of MDSCs in the TME, which induce EMT in tumor cells and increase BC metastasis via upregulating TGF-β1, VEGF, and IL-10 (67). Furthermore, through activating the PI3K-AKT-mTOR pathway, MDSCs promote EMT and boost the production of matrix MMPs in cancer cells, which may boost the invasive and metastatic potential of BC cells (68). M-MDSC from 4T1 tumor-bearing mice with induced tumor site infiltration has been reported to promote tumor metastasis by inducing nitric oxide synthase 2 (NOS2) production and activating STAT1 and STAT3 signaling pathways in tumor cells to induce EMT and CSCs phenotypes (69). Moreover, in an *in vitro* experiment with dog BC cells, MDSCs-secreted IL-28 stimulated STAT3 in tumor cells, inducing EMT and promoting tumor cell migration (70).

In contrast, G-MDSCs from the lung inhibited the EMT and CSCs phenotypes and promoted tumor cell proliferation (69). In a mouse model of spontaneous BC, the investigators found that MDSCs were recruited to the pre-metastatic lung. These MDSCs promoted mesenchymal-epithelial transformation (MET) of metastatic tumor cells within the lung metastases by expressing versican, which blocked TGF-β-Smad2 mother against decapentaplegic 2 (Smad2) pathway-mediated EMT, and in turn, promoted the growth of tumor lesions at the metastatic sites (71).

#### 3.2.3 Angiogenesis

Through stimulating blood vessel growth, MDSCs help contribute to tumor growth, persistence, and dissemination (**Figure 3C**). CXCL17 released by BC cells upregulates MDSCs, which in turn secretes platelet-derived growth factor-BB and promotes the progression and metastasis of BC by establishing angiogenesis in the lung microenvironment (72). Similarly, by secreting IL-28, MDSCs promote tumor cell STAT3 activation and VEGF upregulation, leading to endothelial cell-induced angiogenesis (70). Other studies in mice with revealed that MDSCs produced a large amount of MMP9 (73) and reduced the production of platelet factor 4 (74), resulting in increased permeability and abnormal barrier function of newly generated vessels, which facilitated adhesion of peripheral CTCs to the vessel wall and leakage from the vessel to the metastases.

## 4 Clinical significance of MDSCs in breast cancer

### 4.1 Staging and prognosis

**Figure 4:**
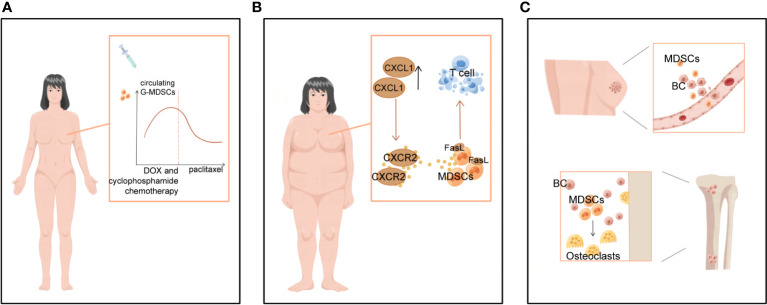
Clinical significance of MDSCs in BC-related staging and prognosis, obesity and bone metastases. **(A)** MDSCs levels varied with treatment progression. In women with operable breast cancer treated with NAT, circulating G-MDSCs increased during DOX and cyclophosphamide chemotherapy, decreased sharply during paclitaxel chemotherapy, and reached near baseline levels at the end of chemotherapy. **(B)** BC-related high-risk factors for obesity contribute to more MDSCs. **(C)** MDSCs not only promote breast cancer bone metastasis, but also differentiate into osteoclasts and cause bone destruction.

Higher levels of MDSCs in BC patients are associated with poor clinical outcomes. META analysis showed a significant association between high levels of MDSCs and poorer estimated OS in BC (6).. MDSCs levels can fluctuate following treatment. Experiments on mice with surgically excised primary cancer demonstrate that surgical stress promotes MDSCs recruitment into lung and tumor tissue (67). Moreover, neoadjuvant chemotherapy (NAT) influences the MDSCs count in TME (74, 75). In women with operable BC treated with NAT, circulating G-MDSCs increased during DOX and cyclophosphamide chemotherapy, declined sharply during paclitaxel, and returned to levels near baseline by the end of chemotherapy (**Figure 4A**). Levels of M-MDSCs were significantly lower, accounting for 1% or less of peripheral blood mononuclear cells, and did not differ significantly over time (76). Similar results were obtained in an experiment in 2022 (75). In a study of BC patients presenting with metastasis or recurrence, M-MDSCs have been observed to greatly expand in the peripheral circulation and associated with greater metastases to LNs and other organs, according to experiments that specifically targeted M-MDSCs. Patients with high levels of M-MDSCs suffer from a more severe condition and an inferior prognosis (77). Tracking M-MDSCs levels in patients with BC could serve as a fascinating and straightforward biomarker for monitoring cancer progression.

### 4.2 MDSCs and breast cancer-related high-risk factors: obesity

Obesity has been linked to an increased incidence of BC, especially in postmenopausal women, and a poorer prognosis of BC disease in all age groups of women patients (78). Obesity and a high-fat diet substantially boost the number of MDSCs in TME, thus further promoting tumor progression (78, 79). Obesity has been reported to increase CXCL1 concentrations in the BC microenvironment, contributing to CXCR2-mediated chemotaxis and the accumulation of G-MDSCs expressing the Fas ligand (FasL). *In vitro* and *in vivo* studies revealed that G-MDSCs cause immunotherapy tolerance by inducing Fas/FasL-mediated apoptosis of T cells (80) (**Figure 4B**). In two mouse models of TNBC, 4T1 and Py8119, glycolytic restriction suppresses G-CSF and GM-CSF expression and decreases MDSCs (81). Furthermore, exercise has been shown to slow the tumors from developing by inhibiting the collection of MDSCs (80–82). This indicates that a healthy diet, weight management, and increased physical activity can also prevent BC by preventing an increase in MDSCs.

### 4.3 Bone metastases and bone destruction in breast cancer

Bone is a significant target for BC metastases, and bone metastases are also a major cause of BC death, usually manifesting as excessive osteoclast-mediated osteolysis, causing fractures and pain in patients (83). During this process, MDSCs suppress the immune system and induce osteolysis in patients with BC by developing into osteoclasts (83, 84) (**Figure 4C**). In particular, MDSCs could only differentiate into osteoclasts under the local microenvironmental conditions of BC bone metastases. In contrast, MDSCs isolated from mice without bone metastases did not undergo such differentiation (83, 85). Studies have revealed that MDSCs cause osteolysis in BC bone metastases by acting as BC osteoclast progenitors and maturing into viable osteoclasts through a NO-dependent mechanism (84, 86). Kun Z et al. found that the tumor-derived integrin ligand epidermal growth factor-like repeat and disc-like structural domain 3 (EDIL3) suppresses tumor-induced MDSCs differentiation into osteoblasts *in vitro* and lowers tumor-induced MDSCs proliferation *in vivo* (86). Thus, treatments that aim specifically at MDSCs may be able to diminish not only their immunosuppressive activity but also MDSCs-mediated osteolysis and the management of BC sequelae.

## 5 MDSCs as a target for breast cancer treatment

As mentioned previously, MDSCs play an irreplaceable role in the progression of BC. Therefore, MDSCs have been identified as a target for tumor immunotherapy. Clinical trials of targeted MDSCs related to BC are summarized in (**Supplementary Table 1**).

Currently, targeting MDSCs includes four main approaches (**Supplementary Table 2**): (1) depletion of MDSCs; (2) blocking the recruitment of MDSCs; (3) suppressing the immunosuppressive function of MDSCs; (4) differentiation of MDSCs to a non-suppressive immune state.

### 5.1 Depletion of MDSCs

First strategy for targeting MDSCs: removal of circulating and tumor-infiltrating MDSCs. It has been demonstrated that low-dose chemotherapy can eradicate MDSCs populations in tumor-bearing rodents; chemotherapies such as gemcitabine, 5-fluorouracil (5-FU) (87), Docetaxel (88) and paclitaxel (89) deplete MDSCs and enhance anti - tumor immune function. However, gemcitabine and 5FU activate the thermal protein structural domain of the NOD-like receptor family containing the 3-protein-dependent inflammasome complex in MDSCs, resulting in the generation of IL-1β. MDSCs-derived IL-1β further stimulates CD4^+^T cell IL-17 production and impairs their anti-tumor activity (87).

Inhibition of VEGF receptor signaling resulted in reduced infiltration of MDSCs. R84 is an anti-VEGF suppressant that blocks the creation of cytokines and chemokines in tumors, especially CXCL1, IL-6 and IL-1β (90). R84 specifically suppresses VEGF binding to VEGFR_2_, which leads to efficient control of tumorigenesis and suppression of suppressive immune cell (MDSCs, Tregs, macrophages) infiltration while boosting the number of mature DCs (90). Anti-angiogenic treatment with the hypoxia-inducible factor-1 (HIF-1) dimerization inhibitor acridine flavin in combination with sunitinib reduced MDSCs accumulation in the spleen, significantly reduced the production of VEGF and TGF-β and retarded the growth of tumors in a mouse model (91). Based on the preceding, we hypothesized that the number of MDSCs could be effectively reduced by targeting VEGF.

Other methods have also been demonstrated to decrease MDSCs. DKN-01 is an IgG4 antibody that specifically neutralizes human and mouse DKK1, a secreted Wnt signaling regulator, and causes a reduction in MDSCs in tumors and spleens and upregulation of PD-L1 on MDSCs in a mice BC model (92). Zonneville et al. found that pharmacological p38 inhibitors (p38i) substantially reduce MDSCs within the TME in TNBC, while depleting MDSCs and simultaneously reducing the expression of chemokines derived from tumors and stroma that are thought to contribute to the recruitment of pro-tumor bone marrow populations (93). Moreover, type I interferon reduces MDSCs in both bone marrow and blood and dramatically reduces bone metastases and metastasis-free survival (94). In addition, a pharmacotherapy liver X nuclear receptor (LXR) agonism decreased the number of MDSCs in a mouse model and in the first human dose-escalation phase 1 trial (95). It has been found that MDSCs have elevated levels of TNF-related apoptosis-inducing ligand receptors (TRAIL-Rs); thus, DS-8273a, a new agonistic TRAIL-R antibody, can induce MDSCs apoptosis and decrease the quantity of MDSCs in the peripheral circulation of BC patients (NCT02076451) (96). In addition, a unique strategy of using spherical nucleic acids encapsulated with antigens encapsulated in lysates containing TNBC cells reduced the frequency of MDSCs in TME, significantly inhibited tumor growth, and prolonged life span (97).

In addition, a number of extensively utilized clinical treatments have been proven to decrease MDSCs. A clinical study showed that resection of the primary tumor in metastatic BC reduced the frequency of MDSCs and improved patient prognosis (98). However, another study showed that resection of the primary tumor in 4T1-bearing mice certainly reduced the number of MDSCs, but did not affect the eventual progression of metastatic BC (99). Furthermore, Habibi M et al. demonstrated that intratumoral injection of IL-7 and IL-15 into mammary carcinoma-bearing mice after radiofrequency thermal ablation reduces MDSCs, induces an immune response to the tumor, and inhibits tumor progression and lung metastasis (100). It has been proven that NKT cell activation therapy could reduce the number and inhibitory activity of MDSCs. However, NKT cell activation in conjunction with either gemcitabine or cyclophosphamide did not further reduce the number of MDSCs (101).

### 5.2 Blocking MDSCs recruitment

The second strategy for targeting MDSCs: blocking the recruitment of MDSCs. As mentioned above, the production and recruitment of MDSCs is regulated by multiple pathways, and targeting and blocking some of these pathways, such as chemokines and CSF, will achieve the goal.

#### 5.2.1 Chemokine

Chemokines serve an essential role in MDSCs recruitment, and a variety of drugs targeting chemokines are currently under investigation. Therefore, MDSCs accumulation can be diminished by inhibiting cytokine or chemokine receptors on MDSCs. In a mouse model of BC, the poly (ADP-ribose) polymerase inhibitor (PARPi) olaparib was shown to inhibit MDSCs migration via the SDF1a/CXCR4 axis (102). In addition, MDSCs express large amounts of CXCR2. ΔNp63 promotes MDSCs recruitment to primary and metastatic sites through transcriptional activation of chemokines, such as CXCL2 and CCL2. The use of inhibitors of CXCR2 (SB 225002) or CCR4 inhibitors (Tocris) or the reduction of transcription factor ΔNp63 levels greatly reduces MDSCs recruitment and the angiogenesis and metastasis it causes (37). Another study demonstrated thatlow dosages of entinostat (ENT) and 5-azacytidine decreased the recruitment of MDSCs by downregulating CCR2 and CXCR2, correspondingly, and extended the OS of mice following surgical excision of the primary tumor (103). Similarly, Silibinin, a natural flavonoid from *Sylibum marianum* seeds, can down-regulate tumor-specific homing of MDSCs by decreasing CCR2 expression on MDSCs (104). Moreover, neutralization of CXCL1 with antibodies was observed to prevent the recruitment of senescent lung fibroblast-mediated G-MDSCs in the MMTV-PyVT recombinant mice (32). Indeed, by targeting the CCR5-CCL5 interaction, the CCR5 antagonist Maraviroc reduced lung metastases in a preclinical mouse model of BC (105). Moreover, AMD3100 is an antagonist for the CXCR4 receptor that diminishes the impact of SDF-1α on MDSCs recruitment, whereas gemcitabine is a drug that eliminates MDSCs directly. The accumulation of MDSCs in the TME is promoted by estrogen-induced production of SDF-1α, an effect which can be inhibited by AMD3100 or gemcitabinem (106)..

Notably, Chinese medicine can also block MDSCs recruitment through various mechanisms. The XIAOPI formula led to a substantial reduction in the quantity of MDSCs in lung tissue by reducing CXCL1 expression in a mouse model of BC (107). Baoyuan Jiedu decoction is a traditional Chinese medicine that inhibits MDSCs recruitment in pre-metastatic niches in the lung via the TGF-β/CCL9 pathway in BC (108).

#### 5.2.2 CSF

CSF-1R is another major target to inhibit MDSCs recruitment to tumor sites to constrain tumorigenesis. Mice with p53N-C tumors had fewer MDSCs after treatment with the mTOR inhibitor rapamycin. Mechanistically, the accumulation of MDSCs is mediated by G-CSF, a downstream target gene of the mTOR pathway. Blocking G-CSF therefore may inhibit MDSCs accumulation (30). In addition, BMP4, a constituent of the TGF-β family, inhibits NF-κB function in human and mouse BC to reduce G-CSF expression (109). Kumar V et al. found that both TAMs and G-MDSCs were diminished at the tumor site by the combination of CSF1R and CXCR2 inhibitors. In contrast to the absence of antitumor activity exhibited by each inhibitor alone, combination therapy drastically decreased tumor growth (110). However, depleting MDSCs with anti-Gr1 or anti-G-CSF antibodies reduced the growth and proliferation of IL-17A-expressing 4T1 tumors but had no impact on control 4T1 tumors (111).

#### 5.2.3 Interleukin

The interleukin family also plays a crucial role in MDSCs recruitment. In TNBC, Bo Yu et al. proved that IL-6 knockdown inhibited SMAD3 K20/117Q -induced MDSCs recruitment (41). In addition, Anakinra, an IL-1 receptor antagonist, was found to reduce MDSCs and M2 macrophage recruitment in a mouse model of mammary tumors with PI, but had no significant effect on M1 macrophages (43). Dominguez C et al. proved that when combined with docetaxel, a clinical-stage mAb that neutralizes the chemokine IL-8 (HuMax-IL-8) was shown to reduce the recruitment of G-MDSCs at tumor sites (112). Curcumin, a substance with IL-6 inhibiting function, reduces the production of IL-6 in BC, thus reducing the number of MDSCs (113).

#### 5.2.4 Others

In addition to the drugs mentioned above, a variety of drugs can inhibit MDSCs recruitment. Leukadherin-1, a allosteric regulatory agent of CD11b, leads to reduced tumor CD11b^+^ MDSCs (114). In addition, AZD4547, a small molecule inhibitor of tyrosine kinases targeting fibroblast growth factor receptors (FGFRs), reduces MDSCs accumulation in carcinoma and lung tissue and suppresses tumor development and lung metastasis (115). The Apelin/Apelin receptor signaling pathway, which is independent from VEGFR signaling pathway, is associated with developmental angiogenesis. It was found that genetic and pharmacological inhibition of Apelin led to a massive decrease in G-MDSCs in tumors, remodeling of TME, reduction of angiogenesis, and effective inhibition of tumor growth (116). Moreover, MIF inhibitor sulforaphane also inhibits MDSCs formation (22). F1 antibody to aspartate protease cathepsin D (cath-D) blocks recruitment of immunosuppressive tumor-associated macrophages M2 and MDSCs and inhibits tumor growth in two TNBC xenografts (117). In a mouse model of BC, PI-3065, an inhibitor of phosphatidylinositol 3-kinase δ (PI3Kδ) signaling enzyme, was able to indirectly inhibit MDSCs recruitment in the TME (118). Atovaquone, an antiprotozoal medication that used treat malaria, decreases the production of ribosomal protein S19 (RPS19) in tumors, thereby decreasing the number of MDSCs and Tregs (119). Markiewski MM et al. discovered that C5aR1 inhibitor (C5aRA) therapy reduced tumor-infiltrating MDSCs and peripheral blood MDSCs in FVB/N Her2/neu transgenic mice (120). Analysis revealed that inhibiting C3a signaling in conjunction with DOX therapy substantially reduced G-MDSCs and eosinophils recruitment, while reducing M-MDSCs recruitment (121).

Above all, the recruitment of MDSCs can be inhibited by a wide range of medications, according to an extensive corpus of research on BC. This indicates that research aimed at inhibiting the recruitment of MDSCs is promising. However, most are still in the experimental phase, and their clinical efficacy remains unclear.

### 5.3 Suppressing the immunosuppressive function of MDSCs

The third strategy for targeting MDSCs: inhibition of the immunosuppressive function of MDSCs. Studies on the suppression of MDSCs activity are plentiful in malignant melanoma and ovarian cancers, but few in BC (122). DC101, an anti-VEGFR-2 antibody, partially diminishes the suppressive effect of M-MDSCs on T-cell propagation and decreases the number of Tregs in both the primary tumor and lung metastases. Remarkably, ARG-1 can be induced by treatment with DC101. The ARG inhibitor N^ω^-hydroxy-nor-Arginine (Nor-NOHA) reduced the inhibition activity of MDSCs on T-cell proliferation and diminished the number and extent of lung metastases, but the combination with DC101 had little additional effect (123). Chaib M et al. proved that protein kinase C (PKC) agonists inhibited the expansion of MDSCs in HPCs and stimulated the differentiation of M-MDSCs into an APC-like phenotype expressing CDC1-related markers by activating the p38 mitogen-activated protein kinase (MAPK) pathway. Functionally, PKC agonists diminished the inhibitory activity of MDSCs and increased their cross-stimulatory capacity (124).

### 5.4 Differentiation of MDSCs to a non-suppressive immune state

A fourth strategy for targeting MDSCs: promoting the differentiation of MDSCs into non-immunosuppressive cells. Effective concentrations of all-trans retinoic acid (ATRA) can eliminate IMCs by neutralizing high ROS production in cells, and induce the differentiation of MDSCs (125). In BC, blocking MDSC expansion with ATRA improves the efficacy of anti-angiogenic therapy (126). Mechanistically, ATRA significantly and specifically upregulates glutathione synthase (GSS) and accumulates glutathione (GSH) in MDSCs, thereby neutralizing the high ROS production in these cells to induce MDSCs differentiation (125). In addition, the application of other chemotherapeutic agents also promotes the differentiation of MDSCs into new cell types that lack immunosuppressive functions. For example, paclitaxel promotes the differentiation of MDSCs into DCs *in vitro* (89); docetaxel inhibits STAT3 phosphorylation and differentiates MDSCs to M1-type macrophages (88).

In addition, various studies have been conducted to promote MDSCs differentiation in BC. Inhibiting the differentiation of M-MDSCs by utilizing ER inhibitors or anti-IL-34 monoclonal antibodies can suppress tumor growth by inhibiting M-MDSCs accumulation and restore tumor chemical sensitivity by promoting G-MDSCs accumulation (21). Svoronos N et al. found that the Jak1/2 inhibitor Ruxolitinib significantly inhibited M-MDSCs differentiation and G-MDSCs expansion, and these inhibitory effects were significantly enhanced by concomitant use of the ERα antagonist methylpiperidino pyrazole (MPP) (127). Moreover, serine/threonine protein kinase CK2 inhibitors, which cause differentiation arrest, significantly reduced the number of G-MDSCs and TAMs (128). Cyclic di-Guanosine (c-di-GMP) is a ligand for interferon gene stimulator (STING). Chandra D et al. demonstrated that low concentrations of c-di-GMP can promote MDSCs maturation to eliminate MDSCs. The antitumor activity of CD8^+^ T cells is enhanced by a Listeria monocytogenes (LM) vaccine expressing the tumor-associated antigen Mage-b (LM-Mb). The combination of LM-Mb and c-di-GMP reduces the effect of MDSCs on CD8^+^ cells and enhances anti-tumor immunotherapy further (129).

Reprogramming the epigenome is an innovative strategy for targeting tumor-promoting properties of MDSCs. In combination with anti-PD-1, anti-CTLA-4, or both, the histone deacetylase inhibitor ENT significantly reduced the suppressive effect of G-MDSCs in the TME, increased CD8^+^ T-effector cells, and ultimately improved tumor-free survival in a mouse model of HER2/neu transgenic BC (130). However, the clinical trial of ENCORE 602 (NCT02708680), which included ENT for TNBC, failed to increase progression-free survival.

As mentioned previously, MDSCs have been shown to be associated with bone metastasis from BC. It has been demonstrated that MDSCs isolated from the tumor-bone microenvironment differentiate into functional osteoclasts both *in vitro* and *in vivo*, with NO signaling as a key regulatory pathway. NG-monomethyl-L-arginine acetate (L-NMMA), an inducible NO synthase (iNOS) inhibitor, inhibited NO signaling and blocked MDSCs differentiation into osteoclasts (85). Interestingly, bisphosphonates, a drug that has been used in BC bone metastases, were discovered to decrease to reduce the number of bone marrow and peripheral blood MDSCs (131). Additionally, the combination of anti-Gr1-mediated depletion of G-MDSCs and zoledronic acid (ZA)-induced osteoclast blockade inhibited the development of established skeletal metastases (132). Therefore, targeted inhibition of MDSCs can not only slow down the course of the primary tumor, but also prevent or delay BC bone metastases simultaneously. Thus, further appropriate clinical studies to back up this conclusion are much anticipated.

### 5.5 Novel drugs

A novel therapy targeting MDSCs based on traditional drugs is currently under development. The new therapy has various advantages, such as more efficient drug delivery and lower incidence of adverse reactions. The relevant new therapies are summarized in **Table 2**.

**Table 2:** Novel therapies based on traditional drugs are being developed.

name	Brief description	Main interaction with MDSCs in breast cancer	Reference
DOX/IND@NPs	A polymeric prodrug nanoparticle based on indocimod with DOX entrapment	reduced the MDSCs	(133)
Tel@PGE	Reduces the proportion of MDSCs in TME and promotes polarization of MDSCs to tumor-killing antigen-presenting cells	Reduces the proportion of MDSCs in TME and promotes polarization of MDSCs to tumor-killing antigen-presenting cells	(134)
BAGEL-R848	A Pluronic F127/hyaluronic acid (HA)-based hydrogel with embedded manganese dioxide (BM) nanoparticles and TLR7 agonist Reximod (R848) for injection.	Reducing the localization of MDSCs in tumors; Inducing *in situ* laser-assisted gelation of hydrogels and achieving the desired ablation temperature within a short laser exposure time	(135)
RLA/DOX/αGC NP	A tumor-targeted c (RGDfk) peptide-modified low-molecular-weight heparin-all-trans retinoic acid (LMWH-ATRA) micellar nanoparticle (RLA/DOX/αGC NP) containing the chemotherapeutic drug DOX and the immune adjuvant α-galactoside ceramide (αGC)	LMWH inhibits the recruitment of MDSCs, while the hydrophobic fragment ATRA promotes MDSC depletion by inducing MDSC differentiation in a whole-brain model of breast cancer	(136)
LMWH-AST/DOX, LA/DOX NP	Colloidal low molecular weight heparin-astatin nanoparticles containing DOX	The hydrophilic LMWH of LA/DOX NPs partially blocked the adherence of MDSCs to VECs and inhibited their recruitment. Hydrophobic AST partially blocked the NF-κB and STAT3 inflammatory signaling pathways in MDSCs and reduced the production of downstream inflammatory factors.AST was effective in reducing ROS levels in MDSCs.	(137)
pCCL2 traps	Plasmid DNA encoding a CCL2 trap	reduced the number of immunosuppressive MDSCs; facilitate PD-L1 blockade immunotherapy	(138)
immuneCare-DISC (iCD)	releasing gemcitabine; cancer vaccines	suppress immunosuppressive MDSCs in the tumor and spleen	(139)
DTX@VTX NP	A nucleus-shell small molecule nanodrugs DTX@VTX NP (VTX: VTX-2337 or Motolimod)	Reversal of the M2 phenotype of immunosuppressed TME to M1 phenotype by depletion of MDSCs; Synergistic action with PD-L1 nano-inhibitors BMS-1 NPs to remodel immunosuppressed TME and enhance the antitumor effect of breast chemoimmunotherapy	(140)
Pseudoneutrophil cytokine sponges (pCSs)	Plasma membrane encasing neutrophils with phenotype and morphology similar to G-MDSCs	Disrupts the expansion of MDSCs and reverses immune tolerance; increases the number of tumor-infiltrating T lymphocytes and restores their anti-tumor function	(141)

### 5.6 Targeted MDSCs in combination with other antitumor therapies

#### 5.6.1 Immunotherapy

Immunotherapy is playing an increasingly important role in cancer treatment. And MDSCs are the essential immunosuppressive cells. Therefore, targeting MDSCs in combination with immunotherapy shows great potential.

Gene-engineered tumor cell derived exosome-like vaccine (eNVs-FAP) developed by Hu S et al. inhibits tumor progression by reducing the number of MDSCs, reprogramming TME and promoting tumor ferrocytosis (142). Another study has shown that a vaccine against fibroblast growth factor (FGF)-2 also reduced the number of MDSCs in mice bearing 4T1 mammary tumors (143). However, an epitope gene vaccine against fibroblast activating protein (FAP) α enhanced the antitumor immune response in BC models, but no improvement in antitumor effects was observed, which may be related to the vaccine-induced elevation of MDSCs (144). Moreover, the development of monoclonal antibodies targeting MUC1 has been reported to counteract the oncogenic effects of MUC1, eliminating MDSCs in TME and ultimately slowing the progression of BC (145). Researches along these lines have a promising future as a tumor vaccine.

Combining tumor vaccines with other therapies has also yielded positive results in research. A triple therapy combines T-cell inducible vaccine with PD-1 antagonist and CD40 agonist mAb leads to a reduction in tumor-infiltrating G-MDSCs and Tregs, further promoting an active CTL response (146). Geng F et al. developed a tumor vaccine targeting both the tumor stromal antigen FAPα and the tumor cell antigen Survivin in BC, which can effectively inhibit tumor progression, and the combination with low-dose DOX to clear induced peripheral MDSCs can further promote the anti-tumor activity of this vaccine (147). Similar results were obtained in another study combining a liposomal vaccine containing E75 (a HER-2/neu-derived peptide) with liposomal DOX (148). Tumor vaccines in combination with chemotherapy, notably DOX, to eliminate the impact of MDSCs on efficacy is, therefore, bright and promising for clinical translation.

#### 5.6.2 RT in combination with other treatments

RT is presently one of the primary cancer therapeutic approaches. RT activates local anti-tumor immunity and non-irradiated, distant site anti-tumor immunity (distant compartment response) (149). Immunosuppressive cell types, such as Tregs, M2-like TAMs, and MDSCs, may be efficiently recruited and expanded by RT.

It is widely established that MDSCs express PD-L1, and RT has been shown to elevate PD-L1 expression in MDSCs. Together, RT and anti-PD-L1 treatment were able to diminish MDSCs accumulation (150). In one trial, triple therapy with RT, PD-1 blockade and PI3Kαδ inhibitors slowed tumor growth by reducing G-MDSCs and increasing cytotoxic CD8^+^ T cells, converting TME to PD-1 blockade or other conditions favourable to ICIs (151). In addition, group V immunomodulatory receptor (VSIR, most widely known as VISTA) is a co-inhibitory receptor, and VISTA blockade depleted MDSCs in 4T1 tumor TME when used with RT, whereas VISTA blockade alone had no such effect. Furthermore, cyclophosphamide plus RT and dual PD-1/VISTA blockade had a superior therapeutic effect. This was connected with the activation of tumor-infiltrating CD8^+^ T cells as well as the reduction of G-MDSCs inside the tumor (152).

Studies have been conducted to explore the effects of RT in combination with a variety of other treatments, but more trials are still required. Ruiz-Fernández de Córdoba B et al. found that using intratumoral α-irradiation ablation in the presence of immunosuppression and CpG inhibitors inhibited the development of mammary carcinoma in mice (153). In an immunocompetent model, leukadherin-1 therapy resulted in a decrease in tumor MDSCs and demonstrated improved antitumor capacity in combination with RT or paclitaxel (114). Moreover, a tyrosine kinase inhibitor, cabozantinib, was found to reduce the number of MDSCs. However, in a preclinical 4T1 BC model, the combination of cabozantinib and RT did not benefit tumor growth control (154).

## 6 Conclusions

These proofs support the theory that the presence of MDSCs is increased in BC patients and that they have a vital position in the immunological resistance phenotype of the disease. Due to the heterogeneity of MDSCs, it is necessary to develop assays that can more accurately characterize subpopulations of MDSCs in BC patients and to measure MDSCs in peripheral circulation and TME at different stages of BC progression in different subtypes to better understand their production, amplification, and function in peripheral blood and TME, and to observe the relationship between MDSCs and BC stage progression. This review will be utilized to support the practical application of research findings and serve as a foundation for the diagnosis and treatment of targeted MDSCs. Through a complex mechanism, MDSCs in BC promote tumor progression and metastasis. Not only do they exert immunosuppressive effects, weakening anti-tumor immunity to promote BC growth and metastasis, but they also diminish the efficacy of other therapeutic approaches. Diverse MDSCs-targeted therapies (as immunotherapy or in combination with conventional therapies, including chemotherapy and RT) are presently being evaluated for their efficacy in BC preclinically in order to maximize its anti-tumor effects. We believe that an improved understanding of the clinical relevance of MDSCs will encourage the research and development of MDSCs-targeted therapies that will enhance the prognosis of BC patients.

## Author contributions

HL, ZW, YZ and YY all contributed to the writing and editing of the manuscript. All authors contributed to the article and approved the submitted version.
