# Supplementary material for: MDSCs in breast cancer: an important enabler of tumor progression and an emerging therapeutic target
Source: Front Immunol. 2023 Jul 3;14:1199273. doi: 10.3389/fimmu.2023.1199273 (PMC10350567; doi:10.3389/fimmu.2023.1199273)
Supplement: Supplementary file 1 [file Table_1.docx]

Supplementary Material

MDSCs in breast cancer: an important enabler of tumor progression and an emerging therapeutic target

Liu Haoyu, Wang Zhicheng, Zhou Yuntao, Yang Yanming*

*Correspondence: Yang Yanming，[yym180@163.com](mailto:yym180@163.com)

Supplementary Table 1 Clinical trials of targeted MDSCs related to breast cancer.

| Rank | NCT Number | Experimentation with MDSCs | Title | Status | Gender | Age | Phases | Study Type | URL |
| --- | --- | --- | --- | --- | --- | --- | --- | --- | --- |
| 1 | NCT04022616 | Isolate unfavorable immune cells MDSC from the peripheral blood or tumors of study subjects and test how strongly they inhibit function of favorable immune cells called Natural Killer Cells. Isolate unfavorable immune cells called MDSC from the peripheral blood of study subjects who plan to start pre-operative chemotherapy for breast cancer to see if MDSC levels can predict who will respond to treatment. Isolate unfavorable immune cells MDSC from peripheral blood of study subjects who receive pre-operative chemotherapy for breast cancer to identify changes in MDSC levels during treatment can predict who will respond to treatment. Isolate lymph nodes from patients with breast cancer undergoing breast surgery to study what types of Natural Killer Cells are present. | Myeloid-Derived Suppressor Cell Function in Breast Cancer Patients | Recruiting | Female | 18 Years and older   (Adult, Older Adult) |  | Observational | https://ClinicalTrials.gov/show/NCT04022616 |
| 2 | NCT00499122 | Correlation Between MDSC Levels and Pathologic Complete Response (pCR) and Non-Responders | NOV-002, Doxorubicin, Cyclophosphamide, and Docetaxel in Women With Newly Diagnosed Stage II or IIIC Breast Cancer | Completed | Female | 18 Years to 120 Years   (Adult, Older Adult) | Phase 2 | Interventional | https://ClinicalTrials.gov/show/NCT00499122 |
| 3 | NCT04913064 | Relative change in levels of circulating MDSCs within the peripheral blood mononuclear (PBMC) compartment | Effects of White Button Mushroom on Inflammation in Obese Postmenopausal Women at High Risk of Breast Cancer | Recruiting | Female | 21 Years and older   (Adult, Older Adult) | Not Applicable | Interventional | https://ClinicalTrials.gov/show/NCT04913064 |
| 4 | NCT03532087 | Change in myeloid cell (M1/M2 Macrophage, MDSC, DC) numbers and function between the baseline biopsy and the surgical specimen. Change in mature and immature myeloid cells (M1/M2 macrophage, MDSC, DC).\|Shift in myeloid cell function. | Study to Identify the Impact of Denosumab on the Immune System in Patients With HER2 Negative Breast Cancer | Withdrawn | Female | 18 Years and older   (Adult, Older Adult) | Phase 2 | Interventional | https://ClinicalTrials.gov/show/NCT03532087 |
| 5 | NCT04796220 | Impact of Focused Ultrasound and Gemcitabine on the Number of MDSCs in Breast Cancer Patients | Focused Ultrasound and Gemcitabine in Breast Cancer | Recruiting | All | 18 Years and older   (Adult, Older Adult) | Phase 1 | Interventional | https://ClinicalTrials.gov/show/NCT04796220 |
| 6 | NCT04352777 | Impact of Endocrine Therapy and Abemaciclib Treatment on Circulating MDSCs in Breast Cancer | Impact of Endocrine Therapy and Abemaciclib on Host and Tumor Immune Cell Repertoire/Function in Advanced ER+/HER2- Breast Cancer | Recruiting | Female | 18 Years and older   (Adult, Older Adult) | Phase 2 | Interventional | https://ClinicalTrials.gov/show/NCT04352777 |
| 7 | NCT02538432 | Impact of EP4 Receptor Antagonist, AAT-007 (RQ-07; CJ-023,423), on Circulating MDSCs in Breast Cancer | Phase II Trial of EP4 Receptor Antagonist, AAT-007 (RQ-07; CJ-023,423) in Advanced Solid Tumors | Withdrawn | All | 18 Years and older   (Adult, Older Adult) | Phase 2 | Interventional | https://ClinicalTrials.gov/show/NCT02538432 |
| 8 | NCT02157051 | Impact of Vaccine Therapy on the Number of MDSCs in Patients with HER2-Negative Stage III-IV Breast | Vaccine Therapy in Treating Patients With HER2-Negative Stage III-IV Breast Cancer | Active, not recruiting | All | 18 Years and older   (Adult, Older Adult) | Phase 1 | Interventional | https://ClinicalTrials.gov/show/NCT02157051 |
| 9 | NCT02479230 | The correlation between vaccine-induced anti-TBVA T-cell immunity and changes in MDSC levels | Type I-Polarized Autologous Dendritic Cell Vaccine With Tumor Blood Vessel Antigen-Derived Peptides in Metastatic Breast Cancer Patients | Completed | All | 18 Years and older   (Adult, Older Adult) | Phase 1 | Interventional | https://ClinicalTrials.gov/show/NCT02479230 |
| 10 | NCT05621837 | define additional MDSC related biomarkers/ identify novel drugs for Myeloid-derived Suppressor Cells (MDSC) blocking in predisposed patients. perform the first survey assessing the link between MDSC and "perceived social isolation", an emerging western social problem recently shown to cause myeloid cell dysfunction and immunosuppression though neuroendocrine circuits. | Quantifying Systemic Immunosuppression to Personalize Cancer Therapy | Recruiting | All | 18 Years to 90 Years   (Adult, Older Adult) |  | Observational | https://ClinicalTrials.gov/show/NCT05621837 |
| 11 | NCT02957968 | Evaluation of myeloid-derived suppressor cells (MDSC) identified in blood samples post-decitabine and post-pembrolizumab compared to MDSC found in blood samples collected at baseline. | Neoadjuvant Pembrolizumab + Decitabine Followed by Std Neoadj Chemo for Locally Advanced HER2- Breast Ca | Active, not recruiting | All | 18 Years and older   (Adult, Older Adult) | Phase 2 | Interventional | https://ClinicalTrials.gov/show/NCT02957968 |
| 12 | NCT02441270 | Impact of Combined Treatment with Radiation and Cyclophosphamide on the Number of MDSCs in Metastatic Breast Cancer Patients | Evaluation of the Immunological Effects of a Combined Treatment With Radiation and Cyclophosphamide in Metastasized Breast Cancer Patients | Withdrawn | Female | 18 Years and older   (Adult, Older Adult) | Early Phase 1 | Interventional | https://ClinicalTrials.gov/show/NCT02441270 |
| 13 | NCT02780401 | Impact of WOKVAC Vaccine Administration on the Number of MDSCs in Patients with Non-Metastatic, Lymph Node-Positive, HER2-Negative Remitted Breast Cancer | Vaccine Therapy in Preventing Cancer Recurrence in Patients With Non-Metastatic, Node Positive, HER2 Negative Breast Cancer That is in Remission | Active, not recruiting | All | 18 Years and older   (Adult, Older Adult) | Phase 1 | Interventional | https://ClinicalTrials.gov/show/NCT02780401 |
| 14 | NCT04941365 | Correlation between MDSC Numbers and Disease Outcome; The Association between MDSC Numbers and Psychological Characteristics in Patients | Quantifying Systemic Immunosuppression to Personalize Cancer Therapy | Withdrawn | All | 18 Years to 90 Years   (Adult, Older Adult) | Not Applicable | Interventional | https://ClinicalTrials.gov/show/NCT04941365 |
| 15 | NCT03719495 | Changes in MDSCs in premenopausal vs postmenopausal women treated with aromatase inhibitors. | Evaluation of Endocrine Therapy Effects of Host Immunity in Early Stage Breast Cancer | Recruiting | Female | 18 Years to 75 Years   (Adult, Older Adult) |  | Observational | https://ClinicalTrials.gov/show/NCT03719495 |
| 16 | NCT03961698 | Efficacy Evaluation of IPI-549 (Eganelisib), a Selective Inhibitor of PI3K Targeting MDSCs, in Combination with First-Line Treatment (Atezolizumabnab + Paclitaxel) for Breast Cancer Patients | Evaluation of IPI-549 Combined With Front-line Treatments in Pts. With Triple-Negative Breast Cancer or Renal Cell Carcinoma (MARIO-3) | Active, not recruiting | All | 18 Years and older   (Adult, Older Adult) | Phase 2 | Interventional | https://ClinicalTrials.gov/show/NCT03961698 |
| 17 | NCT02650635 | Impact of TLR8 Agonist VTX-2337 + Cyclophosphamide on Circulating MDSCs in Recurrent and Stage IV Breast Cancer Patients | TLR8 Agonist VTX-2337 and Cyclophosphamide in Treating Patients With Metastatic, Persistent, Recurrent, or Progressive Solid Tumors | Terminated | All | 18 Years and older   (Adult, Older Adult) | Phase 1 | Interventional | https://ClinicalTrials.gov/show/NCT02650635 |
| 18 | NCT04986852 | Effect of Olinvacimab With Pembrolizumab in Patients Combination Therapy on Circulating MDSCs in mTNBC Patients" | Olinvacimab With Pembrolizumab in Patients With mTNBC | Recruiting | All | 19 Years and older   (Adult, Older Adult) | Phase 2 | Interventional | https://ClinicalTrials.gov/show/NCT04986852 |
| 19 | NCT02432963 | Effect of Vaccine Therapy Combined with Pembrolizumab Treatment on Circulating MDSCs in Previously Treated Refractory Solid Tumor Patients | Vaccine Therapy and Pembrolizumab in Treating Patients With Solid Tumors That Have Failed Prior Therapy | Active, not recruiting | All | 18 Years and older   (Adult, Older Adult) | Phase 1 | Interventional | https://ClinicalTrials.gov/show/NCT02432963 |
| 20 | NCT01929720 | Impact of a Cognitive Behavioral Therapy Targeting Anxiety, Uncertainty, and Insomnia on the Number of MDSCs in Stage IIIB, IIIC, or IV Breast Cancer Patients | Cognitive-Behavioral Intervention for Worry, Uncertainty, and Insomnia for Cancer Survivors | Completed | All | 18 Years and older   (Adult, Older Adult) | Not Applicable | Interventional | https://ClinicalTrials.gov/show/NCT01929720 |
| 21 | NCT03454282 | Impact of Dietary Intervention on Circulating MDSCs in Breast Cancer Patients | Impact of Dietary Intervention on Tumor Immunity: the DigesT Trial | Unknown status | All | 18 Years to 75 Years   (Adult, Older Adult) | Not Applicable | Interventional | https://ClinicalTrials.gov/show/NCT03454282 |
| 22 | NCT02179515 | Impact of Modified Vaccinia Ankara (MVA)-Based Vaccine on Circulating MDSCs in Breast Cancer Patients | Safety and Tolerability of a Modified Vaccinia Ankara (MVA)-Based Vaccine Modified to Express Brachyury and T-cell Costimulatory Molecules (MVA-Brachyury-TRICOM) | Completed | All | 18 Years to 100 Years   (Adult, Older Adult) | Phase 1 | Interventional | https://ClinicalTrials.gov/show/NCT02179515 |
| 23 | NCT02453620 | Impact of Entinostat, Narulitinib Monoclonal Antibody, and Ipilimumab Monoclonal Antibody on the Number of Myeloid-Derived Suppressor Cells (MDSCs) in Peripheral Blood and Tumor Biopsy in Patients with Metastatic HER2-Negative Breast Cancer | Entinostat, Nivolumab, and Ipilimumab in Treating Patients With Solid Tumors That Are Metastatic or Cannot Be Removed by Surgery or Locally Advanced or Metastatic HER2-Negative Breast Cancer | Active, not recruiting | All | 18 Years and older   (Adult, Older Adult) | Phase 1 | Interventional | https://ClinicalTrials.gov/show/NCT02453620 |
| 24 | NCT04616248 | Impact of CDX-301, Radiation Therapy, CDX-1140, and Poly ICLC on Circulating Myeloid-Derived Suppressor Cells (MDSCs) in Patients with Unresectable and Metastatic Solid Tumors | In Situ Immunomodulation With CDX-301, Radiation Therapy, CDX-1140 and Poly-ICLC in Patients w/ Unresectable and Metastatic Solid Tumors | Recruiting | All | 18 Years and older   (Adult, Older Adult) | Phase 1 | Interventional | https://ClinicalTrials.gov/show/NCT04616248 |
| 25 | NCT05082259 | Impact of ASTX660 in Combination with Pembrolizumab (ASTEROID) on the Number of MDSCs in the Tumor Microenvironment of TNBC Patients | ASTEROID: A Trial of ASTX660 in Combination With Pembrolizumab | Recruiting | All | 18 Years and older   (Adult, Older Adult) | Phase 1 | Interventional | https://ClinicalTrials.gov/show/NCT05082259 |

| Supplementary Table 2 MDSCs as a target for BC treatment. | | | |  |
| --- | --- | --- | --- | --- |
| category | | intervention | |  |
| Depletion of circulating and tumor-infiltrating MDSCs | | 5-fluorouracil (5-FU) | |  |
|  |  | Gemcitabine | |  |
|  |  | Docetaxel | |  |
|  |  | Paclitaxel | |  |
|  |  | DKN-01 | |  |
|  |  | Pharmacological p38 inhibitors (p38i) | |  |
|  |  | Type I interferon | |  |
|  |  | Liver X nuclear receptor (LXR) | |  |
|  |  | DS-8273a | |  |
|  |  | Spherical nucleic acids encapsulated with antigens encapsulated in lysates containing TNBC cells | |  |
|  |  | R84 | |  |
|  |  | Hypoxia-inducible factor-1 (HIF-1) dimerization inhibitor acridine flavin in combination with sunitinib | |  |
|  |  | After radiofrequency thermal ablation injection IL-7, IL-15 | |  |
|  |  | Resection tumor | |  |
|  |  | NKT cell activation | |  |
| Blocking MDSCs recruitment | Chemokine | poly (ADP-ribose) polymerase inhibitor (PARPi) | |  |
|  |  | Inhibitors of CXCR2 (SB 225002) | |  |
|  |  | CCR4 inhibitors (Tocris) | |  |
|  |  | Reduction of transcription factor ΔNp63 | |  |
|  |  | 5-azacytidine and entinostat (ENT) | |  |
|  |  | neutralization of CXCL1 with antibodies | |  |
|  |  | Maraviroc | |  |
|  |  | Silymarin | |  |
|  |  | AMD3100 | |  |
|  |  | Gemcitabinem | |  |
|  |  | XIAOPI formula | |  |
|  |  | Baoyuan Jiedu decoction | |  |
|  | CSF | Rapamycin | |  |
|  |  | BMP4 | |  |
|  |  | CSF1R and CXCR2 inhibitors | |  |
|  |  | Anti-Gr1 | |  |
|  |  | Anti-G-CSF | |  |
|  | Interleukin | IL-6 knockdown | |  |
|  |  | Anakinra | |  |
|  |  | HuMax-IL8 | |  |
|  |  | Curcumin | |  |
|  | Others | Leukadherin-1 | |  |
|  |  | AZD4547 | |  |
|  |  | Genetic and pharmacological inhibition of Apelin | |  |
|  |  | Sulforaphane | |  |
|  |  | C5aR1 inhibitor (C5aRA) | |  |
|  |  | F1 antibody to aspartic protease cathepsin D (cath-D) | |  |
|  |  | PI-3065 | |  |
|  |  | Atovaquone | |  |
|  |  | Blocking C3a signaling combined with DOX treatment | |  |
| Suppressing the immunosuppressive function of MDSCs | | N^ω^-hydroxy-desmethylarginine (Nor-NOHA) | |  |
|  |  | PKC agonists | |  |
| Differentiation of MDSCs to a non-suppressive immune state | | All-trans retinoic acid (ATRA) | |  |
|  |  | Docetaxel | |  |
|  |  | Paclitaxel | |  |
|  |  | Estrogen receptor inhibitors or anti-IL-34 monoclonal antibodies | |  |
|  |  | Jak1/2 inhibitor Ruxolitinib | |  |
|  |  | Serine/threonine protein kinase CK2 inhibitors | |  |
|  |  | LM-Mb and c-di-GMP | |  |
|  |  | HDAC inhibitor ENT , In combination with anti-PD-1, Anti-CTLA-4 or both | |  |
|  |  | NG-monomethyl-L-arginine acetate (L-NMMA) | |  |
|  |  | Bisphosphonates | |  |
|  |  | Combination of anti-Gr1-mediated depletion of G-MDSCs with zoledronic acid (ZA)-induced osteoclast (OC) | |  |
| Novel Drugs | | DOX/IND@NPs | |  |
|  |  | Tel@PGE | |  |
|  |  | BAGEL-R848 | |  |
|  |  | RLA/ DOX/αGC NP | |  |
|  |  | LMWH-AST/DOX, LA/DOX NP | |  |
|  |  | PCCL2 traps | |  |
|  |  | ImmuneCareDISC (iCD) | |  |
|  |  | DTX@VTX NP | |  |
|  |  | Pseudoneutrophil cytokine sponges (pCSs) | |  |
| Targeted MDSCs in combination with other antitumor therapies | Immunotherapy in combination with other treatments | Tumor vaccines | Monoclonal antibodies against MUC1 |  |
|  |  |  | Combination therapy with liposomal DOX and liposomal vaccine containing E75, An HER-2/neu-derived peptide |  |
|  |  |  | Vaccine against fibroblast growth factor (FGF)-2 |  |
|  |  |  | Combining T-cell inducible vaccine with PD-1 antagonist and CD40 agonist mAb (triple therapy) |  |
|  |  |  | Epitope gene vaccines targeting fibroblast activation protein (FAP) -α |  |
|  |  |  | DOX pretreatment enhances FAP-α/survivin co-targeting DNA vaccine |  |
|  |  |  | The FAP-α gene-engineered tumor cell-derived exosome-like vaccine (eNVs-FAP) |  |
|  | Radiotherapy in combination with other treatments | RT and anti-PD-L1 combination therapy | |  |
|  |  | Triple therapy with RT, PD-1 blockade and PI3Kαδ inhibitors | |  |
|  |  | Cyclophosphamide plus RT and dual PD-1/VISTA | |  |
|  |  | Ablation with intratumoral alpha‑irradiation combined with inhibitors of immunosuppression and CpG | |  |
|  |  | Leukadherin-1 and RT or paclitaxel | |  |
|  |  | cabozantinib and RT combination therapy | |  |
